# Bioponics in Tomato Cultivation Toward Sustainable Farming: Evaluation of a Circular Tri-Trophic System Incorporating Aquaponics and Insects

**DOI:** 10.3390/plants14182882

**Published:** 2025-09-16

**Authors:** Anastasia Mourantian, Michalis Chatzinikolaou, Maria Feka, Efi Levizou

**Affiliations:** Department of Agriculture Crop Production and Rural Environment, University of Thessaly, GR-38446 Volos, Greece; amourantian@uth.gr (A.M.); mxchatzinik@gmail.com (M.C.); feka.a.maria@gmail.com (M.F.)

**Keywords:** chlorophyll fluorescence, gas exchange, chlorophyll, nitrogen, leaf area, water use efficiency, greenhouse

## Abstract

Bioponics is a promising agricultural system designed to integrate circular economy principles by recovering nutrients from organic waste. In this context we implemented a tri-trophic circular system, where insect larvae fed on crop residues and fruits were processed into insect meal for fish feed. The water used in fish rearing then irrigated tomato crops in an aquaponic setup, closing the nutritional loop. Tomato was cultivated in this system with the aim of thoroughly evaluating its applicability via assessing the dynamics of growth, yield, and functional responses of the crop across three treatments: coupled aquaponics (CAP), decoupled aquaponics (DCAP), and hydroponics (HP, as control). DCAP matched HP in all parameters assessed and even outperformed it in fertilizer use efficiency by 31%. In contrast, CAP showed reduced growth and yield (by 38%) and limitations in photochemical efficiency and photosynthetic performance, likely due to significant deficiencies in potassium and phosphorus (9-fold and 2-fold lower than in HP, respectively). DCAP demonstrated strong potential to achieve similar crop outcomes to conventional hydroponics with enhanced resource efficiency. Overall, adopting the DCAP variant of aquaponics in this circular nutrition system is a promising alternative to conventional hydroponics, supporting a transition toward more environmentally resilient farming practices.

## 1. Introduction

The enhancement of the sustainability of agricultural production systems is an imperative need in a changing world: a growing global population, land and air degradation due to intensive agriculture and extensive chemical use, water scarcity, and ultimately climate change [1]. Under certain conditions, the widespread adoption of circular soilless cropping systems could enhance the sustainability of food production [2]. Soilless systems provide increased productivity with reduced land use and, moreover, in degraded areas, with minimized yet efficient water use [3]. Gruda [4] identified two main goals for soilless systems: (a) optimizing nutrient and water use efficiency; and (b) establishing circular waste flows. Aquaponics, the combined cultivation of fish and crops in a recirculating water system, meets all these requirements [5]. The system provides the plants with nutrients excreted by the fish into the water. The metabolic products of fish enrich the water with nitrogen and other nutrients essential for plant growth. Aquaponics uses fish waste instead of chemical fertilizers and requires about 10% of the water needed for soil cultivation. This makes it a highly sustainable system with a positive environmental footprint [6]. However, the reduced productivity of typical one-loop aquaponic systems has so far prevented their widespread adoption [7,8].

Aquaponics itself requires modification to remain sustainable, yet productive and efficient in addressing the growing human food needs. The enhancement of efficiency and sustainability in aquaponics can be approached from at least two distinct directions. Firstly, it is proposed that the optimization of productivity can be achieved through the adoption of a decoupled aquaponics variant [9,10]. This variant uses fish-derived water to irrigate crops, following the enrichment of depleted nutrients through the application of modest fertilizer amendments. This aquaponics variant has been the subject of recent research, which has demonstrated its comparable efficacy to that of conventional hydroponics [10,11]. The latter is a highly productive system and a growing sector worldwide, because it provides a controlled environment addressing the environmental requirements of crops, along with accurate nutritional feeding that ensures an optimized production output. The relevant literature repeatedly shows that decoupled aquaponics yields productivity levels similar to, and sometimes higher than, those achieved through hydroponics, and this holds also for high-nutrient-demanding crops such as cucumbers and tomatoes [11,12,13].

The second aspect of aquaponics that requires improvement in order to achieve increased sustainability is the composition of fish feed. The reliance on fish meal, a primary component of fish feed, poses a significant ecological concern and is crucial for the sustainability of aquaponics [14]. Fish meals originate from wild fish populations, although a partial replacement with fishery by-products has been successfully tested [15]. The incorporation of alternative proteins in the nutrition of farmed fish has emerged as a hotspot in the relevant research [16,17]. A promising alternative is the utilization of insect meal, which has been demonstrated to address the nutritional requirements of fish in terms of amino acids and micronutrients [18,19]. Additionally, the insect rearing process is simple and economically viable, with minimal space and feed requirements [20]. This approach is regarded as highly sustainable in cases where agricultural side streams and food processing by-products are incorporated into the insects’ diets [21,22].

We present a circular tri-trophic system that closes the nutritional loop between fish, crops, and insects by incorporating the above-mentioned systems and alternatives. The aim is to achieve a highly productive cropping setup. Fish metabolic products fertilize the crops, while pruning material and non-commercial fruits serve as insect feed. The latter is transformed into insect meal and added to the fish feed. In this way, the waste of one organism becomes a resource and food for the next. Thus, the operation of this tri-trophic system is consistent with the concept of a circular economy, enabling the valorization of waste through the recovery of nutrients that would otherwise be discharged.

In the present study we evaluated the effects of this circular tri-trophic system on tomato cultivation. We reared *Hermetia illucens* (black soldier fly, BSF), which eats virtually everything, and we fed its larvae with tomato fruits and old leaves that were removed from the crop cultivation. The insect meal was then incorporated into tilapia fish (*Oreochromis* sp.) feed. Tilapia and tomato plants were connected via coupled and decoupled aquaponics, with hydroponics employed as a control. This study aimed to evaluate the system’s operation and performance in detail, with three objectives: firstly, to assess the system’s efficacy in terms of crop yield; secondly, to conduct a thorough assessment of the crops’ functional and growth responses; and thirdly, to explore metrics pertinent to the efficiency of fertilizer and water utilization.

## 2. Results

The water quality parameters of the three irrigation solutions are presented in Table 1. DCAP and HP exhibited nearly identical values for all measured physicochemical parameters. The pH of CAP was significantly higher, while the EC was lower compared to the two other treatments, due to fertilizer added to them. The NO_3_^−^ and PO_4_^3−^ concentrations in DCAP and HP were almost double those in CAP, while the [K] concentration in the latter was 122 times lower. Similarly, the Na and Ca concentrations in CAP were found to be 12 and 22 times lower than in HP and DCAP, respectively.

**Figure 1:**
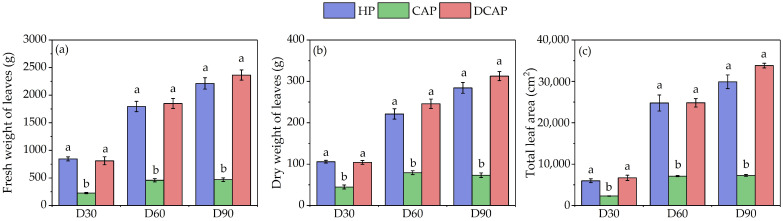
The growth-related parameters of tomato plants at three harvests during the experimental period; fresh (**a**) and dry (**b**) weight of leaves, and total leaf area (**c**), presented as Avg ± SE (n = 10). Different letters indicate statistically significant differences among treatments at each harvest (*p* ≤ 0.05).

**Figure 2:**
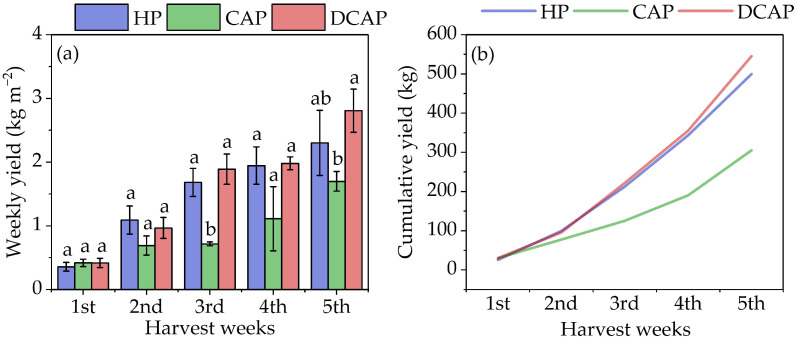
(**a**) The weekly yield in tomato fruits for the three treatments, expressed as kg m^−2^ (Avg ± SE); the different letters indicate statistically significant differences among treatments at each harvest (*p* ≤ 0.05). (**b**) Cumulative yield of tomato fruits for the three treatments throughout the cultivation period, expressed in kg.

Growth performance of tomato plants is presented in Figure 1 for all three harvests. Similar values between HP and DCAP appeared for leaf fresh weight in all recordings, which increased over time (Figure 1a). CAP exhibited an opposite growth pattern, demonstrating significantly inferior fresh aerial biomass accumulation compared to HP and DCAP from the first month of cultivation (D30). Additionally, a negligible increase in fresh weight over the duration of the experiment was evident. Notably, by the end of the experiment (D90), HP and DCAP had approximately five times greater fresh biomass than CAP. As demonstrated in Figure 2b, a similar trend is evident in leaf dry weight, with CAP exhibiting a fourfold reduction in dry biomass compared to the other two treatments. Figure 1c shows the total leaf area (cm^2^) of plants. Since the rate of increase in the CAP leaf area was negligible throughout the experiment, the differences recorded at its conclusion equaled those of fresh biomass. That is, the HP and DCAP values were found to be 4.5 and 5 times greater, respectively.

Figure 2 presents the weekly and cumulative yield of the fruits for the three treatments. Weekly yield is expressed in kg per m^2^ over the 5 weeks of the fruit harvest period (Figure 2a). A steady increase in the weekly yield was observed for all three treatments during the growing period. During the first two weeks of harvest, all treatments exhibited similar performance with no statistically significant differences. After the third week, HP and DCAP continued to exhibit comparable yields. However, DCAP displayed a substantial increase and surpassed HP by the end of the experiment, yet the difference was not statistically significant. CAP, on the other hand, demonstrated a more moderate increase in yield over time, remaining the least efficient of the three treatments. Figure 2b depicts the steady increase in the cumulative yield for the three treatments, with the yield here expressed as kg produced in each treatment. Upon the conclusion of the five-week harvest period, the cumulative production reached 545 kg for DCAP, 499 kg for HP, and 305 kg for CAP.

**Figure 3:**
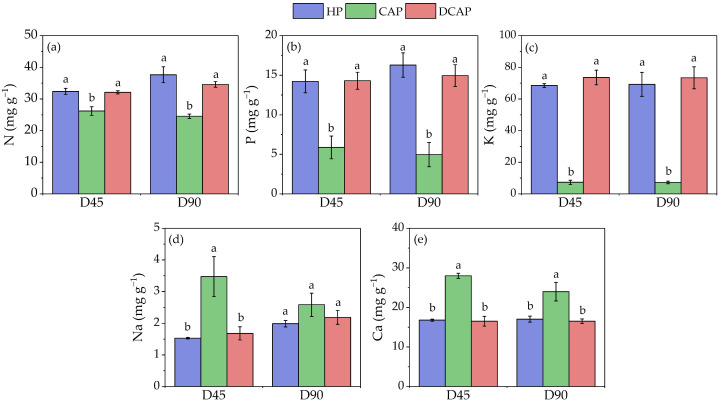
The concentration of (**a**) N, (**b**) P, (**c**) K, (**d**) Na, and (**e**) Ca in tomato leaves at two measurements during the experimental period (Avg ± SE) (n = 6). Different letters indicate statistically significant differences among treatments at each measurement date (*p* ≤ 0.05).

Leaf elemental analysis was performed at the middle and end of the experiment, on D45 and D90 (Figure 3). The concentrations of all nutrients were found to be similar for HP and DCAP, with these being superior to those of CAP plants. However, an exception was observed in the latter, regarding the Na and Ca. CAP leaf concentrations of N were 19% and 35% lower compared to the control (HP), on D45 and D90, respectively. Decreases in P were more significant, with CAP concentrations being almost twofold lower on both measurement dates. However, the K content of CAP leaves showed the most pronounced difference, being 9-fold lower than HP on both measurement dates. On the contrary, an increase in the leaf content of both Na and Ca was observed in the case of CAP, except for D90 for Na. Notably, the [Na] of CAP leaves increased by 127% compared to the other two treatments on D45.

**Figure 4:**
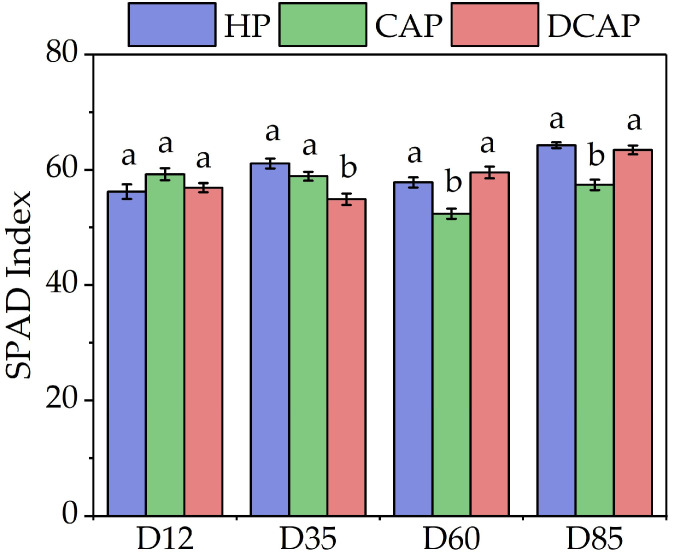
SPAD index measured during the experimental period (Avg ± SE) (n = 10). Different letters indicate statistically significant differences among treatments at each measurement date (*p* ≤ 0.05).

The SPAD index displayed high values and moderate fluctuations during the experimental period (Figure 4). However, statistically significant differences were observed among the treatment groups on three out of four measurement dates. DCAP showed a marginal, yet significant 10% decrease compared to the other two treatments on D35. In the subsequent two measurements (D60 and D85), CAP demonstrated an analogous decrease in SPAD values (10%).

**Figure 5:**
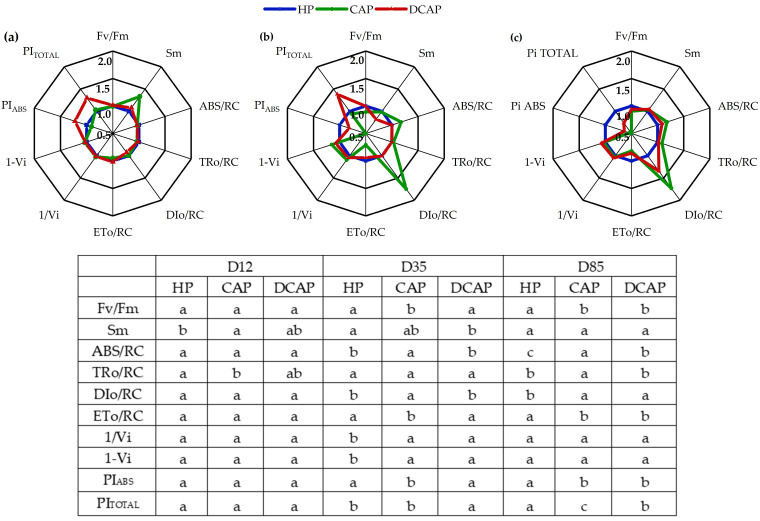
Chlorophyll a fluorescence parameters presented in radar plots for (**a**) D12, (**b**) D35, and (**c**) D80 (n = 15). The values were normalized to HP values (regarded as 1.0). The statistical analysis is presented in the table, where the different letters indicate statistically significant differences among treatments at each measurement date (*p* ≤ 0.05).

The chlorophyll a fluorescence parameters derived from the OJIP test are depicted in the spider graphs (Figure 5). The results of the statistical analysis are presented in the subsequent table. On D12, the only statistically significant difference observed was in the Sm and TRo/RC parameters between the CAP and HP treatments. In the subsequent measurement on D35, CAP exhibited a statistically significant reduction in the Fv/Fm parameter, accompanied by an increase in Dlo/RC and ABS/RC when compared to DCAP and HP. Additionally, HP performed poorly in the parameters related to PSI (1-Vi and 1/Vi), while DCAP recorded a significantly higher value in the Pi_TOTAL_ index. The most significant statistical differences were observed in the final measurement on D85. Specifically, HP outperformed CAP and DCAP regarding the parameters Fv/Fm and ETo/RC and the indices PiABS and Pi_TOTAL_, as evidenced by statistical analysis. Conversely, CAP exhibited an increased value in the parameter DIo/RC. In contrast, the ABS/RC exhibited variation among the treatments, elevated in CAP and diminished in HP.

**Figure 6:**
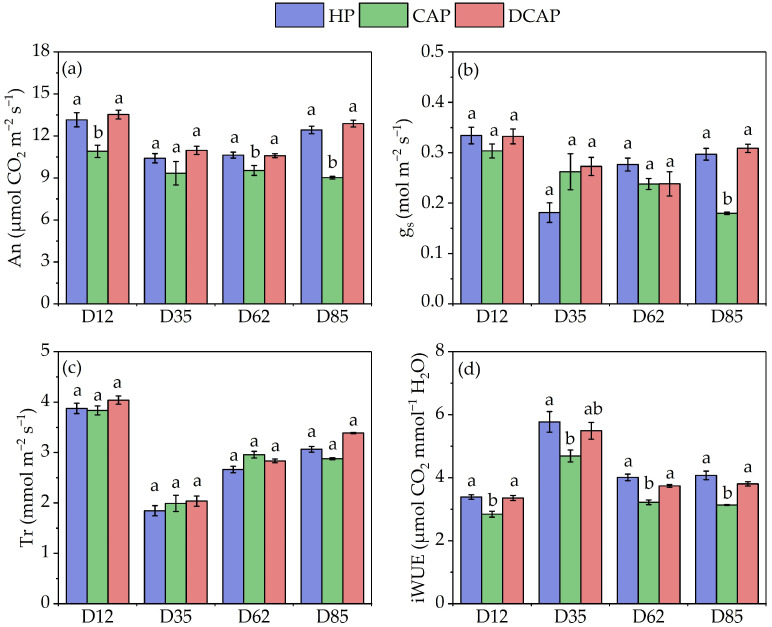
Gas exchange parameters assessed at four time points during the experimental period: (**a**) An, net photosynthetic rate; (**b**) gs, stomatal conductance; (**c**) Tr, transpiration rate; (**d**) iWUE, intrinsic water use efficiency (Avg ± SE) (n = 10). Different letters indicate statistically significant differences among treatments at each measurement date (*p* ≤ 0.05).

Figure 6 depicts the dynamics of gas exchange parameters, An, gs, Tr, and iWUE, monitored on D12, D35, D62, and D85. As demonstrated in Figure 6a, the An generally exhibited values of approximately 10–15 μmol CO_2_ m^−2^ s^−1^, with DCAP and HP demonstrating comparable rates. However, CAP exhibited significantly lower rates than the other treatments, except for on D35, when no statistically significant differences were observed. Figure 6b shows that gs ranged from approximately 0.2 to 0.35 mol m^−2^ s^−1^. Although moderate fluctuations were evident during the experimental period, the only statistically significant difference was recorded on D85, with CAP values showing a 40% decreased gs compared to the other two treatments. Tr was elevated in the initial measurement, yet it was comparable (approximately 4 mmol m^−2^ s^−1^) across all treatments (Figure 6c). On D35, a marked decrease in transpiration rate was observed, yet the values remained constant (approximately 2 mmol m^−2^ s^−1^) across all three treatments, with no statistically significant differences. This profile was similar to that observed on D62 and D85. As demonstrated in Figure 6d, the intrinsic water use efficiency exhibited comparable values across all measurement days, with HP and DCAP demonstrating higher values than CAP. These differences were found to be statistically significant.

DCAP had the highest WUE at 15.91 kg m^−3^, which is a 7.3% increase compared to HP, the control of the experiment (Table 2). Conversely, the lowest recorded WUE was observed in CAP (11.27 kg m^−3^), indicating a 24% decrease compared to HP. FUE calculation was only conducted for HP and DCAP, because the CAP treatment did not involve the addition of chemical fertilizers. The FUE was higher in DCAP, with its value reaching 16.30 kg of tomato produced per kg fertilizer used. This represents a 31.44% increase over the HP value of 11.17 kg kg^−1^.

## 3. Discussion

The cyclic flow of water and nutrients in primary production systems ensures resource saving and reduces the environmental footprint, thereby increasing their sustainability [23,24]. Nevertheless, these advantages must be accompanied by high productivity potential in order to be adopted in highly intensive agriculture. In the circular tri-trophic system implemented in the present study, which exemplifies bioponics in tomato cultivation, we achieved nutrient recycling in a closed-loop system. Thus, we recovered nutrients from material that would otherwise be considered organic waste. We recorded the dynamics of plant responses throughout the experimental period and will analyze them below.

There is a cause-and-effect relationship between the concentrations of nutrients in the irrigation water and the nutritional state of the plants, as well as their growth, yield, and functional parameters [9,25]. More precisely, the availability of nutrients in the aquaponic water exclusively shapes the leaf nutritional status, which subsequently affects the growth and function of crops in combination with environmental greenhouse conditions [26,27]. The CAP irrigation water, which was fully derived from fish tanks, was deficient in all recorded nutrients. While the concentrations of N and P were almost half of those in HP and DCAP, the concentration of K in CAP was found to be 122 times lower. This was the most extraordinary difference recorded, though [Na] and [Ca] were also 12 and 22 times lower, respectively. This remarkable deprivation of essential nutrients has been repeatedly reported in coupled aquaponic systems and has been attributed to the levels of these nutrients in the fish feed, as this is the only route through which they enter the system [28]. This deficiency has prompted significant research in the past two decades, with a focus on identifying effective nutrient management strategies for successful aquaponic production systems [29,30]. After conducting trials involving excessive fertilizer use, this line of research resulted in decoupled aquaponic systems [13,31]. This variant of aquaponics has the potential to address nutritional needs while maintaining equilibrium in the amendments and ensuring the sustainability of the system [11]. In the present experiment, the DCAP treatment achieved comparable nutrient levels to HP, thus achieving high nutrient bioavailability with a 26% reduction in fertilizer amount compared to HP.

The similar and high availability of nutrients in the DCAP and HP irrigation solutions resulted in a comparable leaf nutritional state. Conversely, CAP leaves experienced a significant decrease in [K], which was nine times lower than in the other two treatments. Additionally, decreased levels of [P] were also evident, with a twofold change compared with HP and DCAP. N was reduced in CAP as well, albeit to a lesser extent. Interestingly, the concentration of Na and Ca in CAP leaves was significantly higher than in DCAP and HP leaves, with an increase of 127% in [Na] on D45. Yang and Kim [29] corroborate these findings as they reported six times higher [Na] in the fish water of coupled aquaponics than in a hydroponics control, which resulted in three times higher concentrations in the edible tissues of tomato and basil. The [Na] and [Ca] profiles indicate bioaccumulation, since their concentrations in the irrigation solution were considerably lower than in DCAP and HP. This result corresponds to the well-documented enhancement of inorganic cations such as Na^+^ and Ca^2+^, as well as Mg^2+^ in plants facing K deficiency [32]. The underlying mechanisms appear to differ for Ca and Na. Ca likely replaces K in certain physiological processes, including the activation of some enzymes, cellular pH adjustment, and osmoregulation [33,34]. Regarding [Na], it has also been demonstrated that it replaces K in processes including, but not limited to, cation–anion balance, water relations, membrane and chlorophyll stabilization, and osmoregulation [35]. Conclusively, the increases in [Na] and [Ca] found in the present study may be related to K deficiency in CAP leaves and may alleviate its effects, as discussed below in the section on growth and functional crop responses.

Sub-optimal levels of K, as well as P, are considered responsible for the growth and yield inferiority of CAP tomato plants compared to HP and DCAP. The latter two again showed similar levels in all assessed growth parameters (Figure 1). Their fresh biomass accumulation was five times higher than CAP’s, which showed a stunted growth, as recorded in the dynamics of all measured parameters. Interestingly, the impeded development of leaf biomass and area was apparent by the end of the first month of the experiment. High-nutrient-demanding crops like cucumber and tomato typically show a pronounced growth impairment [11,29] compared to low-demanding ones, e.g., lettuce and basil [9,36] yet in all of them, a negative impact of CAP is evident. K deficiency may account for this growth response, since it may result in reductions in both leaf number and leaf size [34]. The latter has been attributed to perturbed water relations due to low [K], which linearly affect the turgor pressure, high levels of which are necessary for cell elongation [37]. Nevertheless, the poor nutritional state of CAP leaves may also impact growth in other ways. For example, [P] in the form of Pi has been reported to influence growth through interactions of its homeostasis with other nutrients such as Fe and Zn [38]. Thus, in aquaponics research, the addition of K and P has been documented to alleviate the negative effects on growth and restore high biomass accumulation and yields [25,39,40].

The tomato yield in DCAP slightly surpassed that of HP over the entire cultivation period. These similar dynamics in tomato production were obvious from the first harvest week. CAP yielded comparable results until the second week of fruit harvest, after which reduced productivity became evident. Notably, the cumulative yield of CAP was 39% lower than that of HP. Similar results have been reported by Aslanidou et al. [11] when working with the same treatments in cucumber (40% reduced CAP yield); however, in tomato, they found a 50% reduction in CAP compared to HP. They confirmed the slightly better performance of DCAP than HP, which was also found in our work. This result is not rare in the relevant literature and has been associated with the possible presence of microorganisms that promote growth in the fish water used to prepare the DCAP irrigation solution, yet the exact mechanisms remain unclear [41,42,43].

The response of chlorophyll concentration dynamics to the nutritional imbalances imposed by various aquaponic systems is species- and system-specific. In the present study, high and nearly constant levels of total chlorophylls, as determined by the SPAD index, were evident throughout the experimental period. However, only in the last month did CAP plants experience a slight (10%) decrease in the SPAD index compared to DCAP and HP (Figure 3). Tsoumalakou et al. [25] reported a destructive chlorosis of spinach leaves, as early as D12 of their experiment, in coupled systems. Lettuce, which is less sensitive, has shown either no effect [36] or a moderate 15% decrease [8] in CAP treatments.

The temporal dynamics of the functional response of tomato plants to the three tested irrigation solutions were monitored, with a focus on the photosynthetic process. The performance of the photosynthetic apparatus was recorded using the chlorophyll fluorescence technique, which is considered a reliable and powerful tool for analyzing aspects of photosynthetic electron transport and events in the photosystem II (PSII) and PSI activity [44]. DCAP plants showed a superior PSII performance, as indicated by their higher total photosynthetic efficiency index (PI_TOTAL_) as well as PI_ABS_, which signifies enhanced conservation of energy acquired from the photons absorbed by the PSII antenna (Figure 5). This effect was particularly evident on D12 and D35; however, the trend reversed in the final measurement, possibly due to the effects of leaf aging. The results also suggest that the impact on PSI performance was marginal for all dates and treatments according to 1-Vi, indicative of the yield of PSI reaction centers and the 1/Vi parameter, which refers to the yield of PSI final acceptors. DCAP exhibited similar behavior to HP in all other fluorescence parameters. The photosynthetic apparatus of CAP plants showed signs of inferior performance, as indicated by the lower maximum quantum yield of PSII photochemistry (Fv/Fm). Issues in PSII activity were also indicated by the energy flux indices per active reaction center (RC): a remarkable increase in thermally dissipated energy (DIo/RC) after the first month of the experiment, along with a consistent increase in ABS/RC and TRo/RC, absorbed and trapped energy per RC, respectively. These energy flux results may originate from enhanced inactivation of PSI RCs, which has been reported to be associated with nutrient deficiencies [8,45]. This is exemplified by the report of Kalaji et al. [30], working on multiple nutrient deficiency schemes in tomato, who stated that decreased photochemical efficiency and number of active PSII RCs were evident in all the nutrient-deficient plants.

The good performance of the photosynthetic apparatus of DCAP and HP was also obvious in the dynamics of gas exchange parameters. High and stable An was recorded throughout the cultivation period for these treatments, in contrast to CAP, which experienced a 27% decrease compared to HP at the final measurement. Combined with the unaffected Tr, the fluctuations in An rendered a slightly but significantly reduced iWUE in CAP plants. Similar effects on gas exchange are often reported in the relevant aquaponics literature [9,25,36] and have been attributed to the poor nutritional state of CAP leaves. However, when we compare our results with the findings of studies on severely deficient tomato plants of other studies [30,46], a distinct picture arises: the effects of CAP treatment on photosynthetic performance, in terms of both photochemical efficiency (fluorescence data) and actual CO_2_ assimilation, are moderate and do not point toward damage to the photosynthetic apparatus. As in other works of our group, we can attribute this to a modification of the photosynthetic metabolism and activity in order to adapt to low nutrient contents rather than an impairment [36]. In particular, K deficiency has been associated with reduced sink activity in the consumption of photoassimilates, primarily due to K’s pivotal role in carbohydrate translocation and metabolism [34,46]. This reduced sink activity results in feedback inhibition of source activity, which eventually downregulates photosynthesis.

The resource use efficiency of the examined treatments was superior under DCAP. This is valid not only for WUE, which differed minimally from HP, but more remarkably for FUE. The latter showed a pronounced 31% increase compared to the conventional hydroponics, reflecting both an increased fruit yield and a reduced fertilizer amount used in DCAP by 26%. Analogous increases in FUE have been reported in Aslanidou et al. [47] for tomato, basil, and cucumber, while an extraordinary 180% increase in DCAP FUE was reported by Chatzinikolaou et al. [26], working with the less-nutrient-demanding lettuce. These results on resource use efficiency establish DCAP as a sustainable cropping system that remains productive at levels comparable to conventional hydroponics while consuming less fertilizer.

## 4. Materials and Methods

### 4.1. Experimental Setup

The experiment took place in the pilot-scale aquaponic greenhouse of the University of Thessaly, in Velestino (39°440′, 22°790′, altitude 85 m), Greece. The greenhouse’s total area is 440 m^2^, with 360 m^2^ dedicated to crop cultivation and the remaining 80 m^2^ accommodating the fish rearing system within a controlled-condition chamber. The greenhouse roof-covering material is low-density polyethylene, which is resistant to high temperatures and impermeable to water and water vapor. A more detailed description of the pilot aquaponic greenhouse setup and climate management can be found in [47].

#### 4.1.1. Recirculating Aquaculture System (RAS)

The recirculating aquaculture system (RAS) included the following components: (i) three fish tanks with capacity of 1300 L each; (ii) a 650 l buffer tank; (iii) a mechanical filter (Rotary Drum Filter, ProfIDrum B.V., Scandia, MN, USA); (iv) a biofilter of 700 L, containing ceramic rings and K1 media (Kaldnes, 1 mm), which serve as substrates for the proliferation of nitrifying bacteria (Prodibio, Biodigest; Marseille, France); and (v) a 2500 L sump tank. A system of sensors for continuous monitoring of water quality parameters, including temperature, pH, electrical conductivity (EC), and dissolved oxygen (DO), was installed in the sump tank, where the fish water was collected (GHM-Greisinger, pH/EC/O_2_ measuring transducer, Senseca Germany GmbH, Remscheid, Germany). In the three fish tanks, the DO was maintained at 7.0 mg L^−1^ (±0.3 mg L^−1^), using air diffusers and an air blower, while the temperature was kept at 23 °C (±0.4) using temperature regulators [26]. In the RAS, a continuous water flow rate of 6 m^3^ h^−1^ was maintained via a pump (Aquastrong Company LTD, Milan, Italy). The uneaten feed and fish feces from the fish tanks were siphoned out daily. The solid effluent that remained was extracted by the pump of the mechanical filter.

Prior to the start of the experiment, the tilapia fish were weighed and distributed into three tanks based on their size. In total, the rearing system included 238 fish with a total biomass of 117.6 kg. Specifically, the first tank contained 110 fish weighing up to 600 g, with an average weight of 400 g; the second tank housed 71 fish up to 950 g (average 525 g); and the third tank held 57 fish up to 1 kg, with an average weight of 670 g. The initial biomass per tank was 40.1 kg, 39.3 kg, and 38.2 kg, respectively. The fish were fed daily with a fixed amount of feed equivalent to 1% of their body weight. This rate was chosen because it balances covering all the metabolic needs of adult fish (which usually consume 1–1.5% of their body weight) and preventing food waste. It should be stated here that the fish used in this experiment were big, although smaller fish could be tested. However, the latter poses the necessity for additional tanks and reduction in fish density. This is due to the uneven growth patterns exhibited by fish, which give rise to hierarchy issues. From a management perspective, this would result in more challenging circumstances.

#### 4.1.2. Insect Rearing

BSF mating took place within a love cage (70 × 70 × 86 cm) that was illuminated by an LED lamp. Oviposition took place in shady areas of the cage. The collected eggs were transferred to small containers filled with chicken feed to meet the initial nutritional needs of the newly hatched larvae. After one week, the larvae were relocated to a larger container and fed with tomato leaves and fruits. Upon attaining the optimal size, the majority of the larvae were harvested, subjected to oven-drying, and processed into insect meal for utilization in the formulation of fish feed. The rearing facility had a capacity of more than 9.5 kg of fresh larvae, which hatched from 3 g of eggs within a 5-day period (more details in [26]). During the experiment, approximately 75 kg of fish feed was produced, containing 7.5 kg of insect meal (10%), which corresponds to 26.4 kg of fresh larvae. Further details on insect meal preparation and proximate analysis of fish feed can be found in Chatzinikolaou et al. [36].

#### 4.1.3. Crop Cultivation and Experimental Design

The crop cultivation area was occupied by a hydroponic system consisting of 18 channels, each 8.5 m long, 0.22 m wide, and 50 cm high. Drip irrigation (2 L h^−1^) and climate management were automatically controlled through climate control software (Emmanouilidis, Thessaloniki, Greece) with the assistance of temperature, humidity, and solar radiation sensors. More specifically, an air heater provided heating when the temperature dropped below 18 °C. Cooling, with a set point of 26 °C, was achieved through a fan and pad evaporative cooling system. Ventilation was ensured by opening the roof windows when the temperature and humidity exceeded 21 °C and 85%, respectively.

In the present experiment, 276 tomato plants (Solanum lycopersicum) of the OPTASIA × EMPERADOR variety, bearing 5–6 true leaves, were transplanted in perlite bags at a density of 1.76 plants m^−2^. With this particular variety and the prevailing environmental conditions, crops started to produce fruit early (first harvest on D54); thus, the tomato cultivation lasted for 90 days.

**Figure 7:**
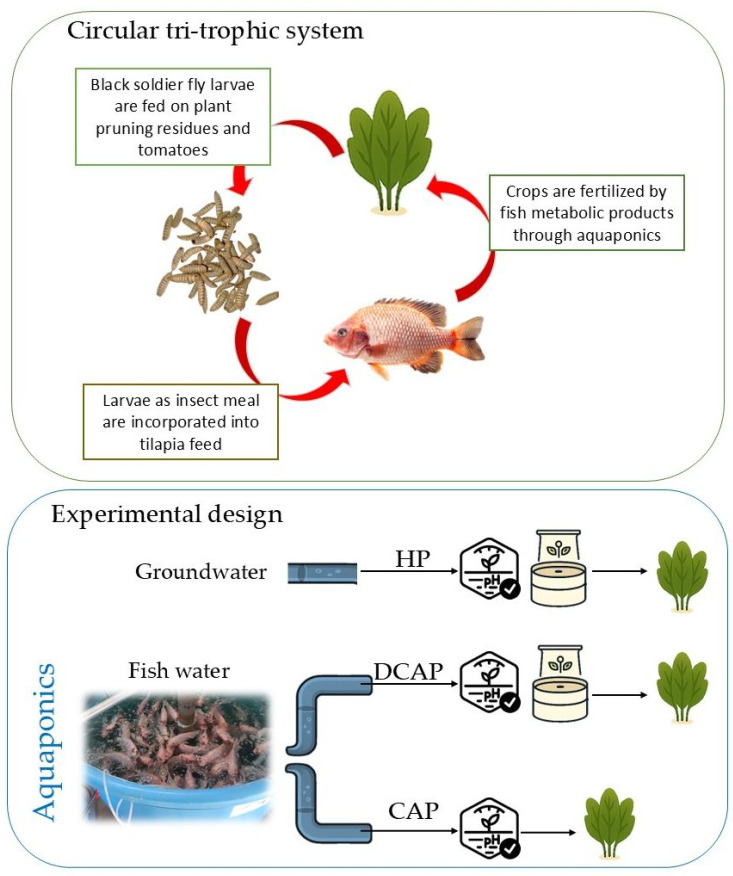
The basic concept of the tri-trophic system (**above**) and the experimental design with the three treatments (**below**).

The treatments involved administering three different nutrient solutions as follows (Figure 7): (a) conventional hydroponic solution (HP), used as the control; (b) aquaponic solution enriched with nutrients to achieve the concentration targets in HP (decoupled aquaponics, DCAP); and (c) aquaponic solution without the supplementation of any nutrient (coupled aquaponics—CAP).

The hydroponic nutrient solution was prepared by mixing inorganic fertilizers according to the formulation designed by Savvas et al. [48], which is designed for hydroponic tomato cultivation under Mediterranean conditions (Table 3). The nutrient concentrations in the DCAP solution were determined through weekly monitoring of the fish tank water. Subsequent adjustments were made through chemical fertilizer amendments to align the nutrient concentrations with those of the HP solution [26].

The crop cultivation area was divided into three blocks, each containing 6 hydroponic channels, which were randomly allocated to the three treatments. This configuration resulted in 6 replications of each treatment (2 channels per block × 3 blocks), giving a total of 92 plants/treatment.

### 4.2. Physicochemical Parameters of the Irrigation Solutions

The physicochemical parameters of the water in the three irrigation solutions were followed throughout the experimental period. The pH and EC were measured using a portable sensor (HQ40d, Hach, Loveland, CO, USA). The pH was maintained at 5.6 for all treatments; nitric acid (HNO_3_ 65%) in combination with sulfuric acid (H_2_SO_4_ 96%) was used for the pH adjustment in HP and DCAP treatments, while only sulfuric acid (H_2_SO_4_ 96%) was used for CAP to avoid adding nitrogen to this treatment. The electrical conductivity (EC) levels were adjusted at 2.5 dS m^−1^ by the central mixing tank (the head unit), in which the irrigation solution was prepared. This EC value was set according to the recipe used for the tomato cultivation. The concentration of nutrients and specifically of NO_3_^−^, PO_4_^3−^, K^+^, Ca^2+^, and Na^+^ in the three irrigation solutions was measured according to Aslanidou et al. [47].

### 4.3. Crop Measurements

#### 4.3.1. Plant Growth and Yield

During the experiment, growth measurements were conducted in the plants, through three destructive harvests (plant cuttings), which were carried out on days 30, 60, and 90 (D30, D60, and D90). During each harvest, the aerial part of 10 plants/treatment was cut, and the fresh weight of the leaves was immediately measured. The total leaf area was assessed with a leaf area meter (LI-COR 3100C, LI-COR Environmental, Lincoln, NE, USA). Subsequently, the leaves, without any additional handling such as washing, were transferred to 70 °C conditions until a constant weight was achieved, after which the dry weight was recorded.

The yield of the crops of each treatment was measured on a weekly basis, with the first recording being performed on D54. Tomato fruits of commercial size were harvested every 2–3 days, collected separately for each treatment. Immediately after harvesting, the fruits were weighed to determine their fresh weight. The total weight of the sum of fruit collected each week from each treatment was considered the “weekly yield” and is expressed in kg/m^2^ (presented in Figure 2a). Since there were three blocks, each block was considered a replication unit; thus, the weekly yield assessments in Figure 2a are presented as the average and standard error (SE) of three replicates, alongside the corresponding statistical results. The cumulative fruit yield (Figure 2b) was calculated by adding together the current and previous weekly fruit yields for each treatment and is expressed in kg.

#### 4.3.2. Leaf Nutrient Concentration

The leaf elemental analysis was performed on dry samples collected at the middle and end of the experiment (D45 and D90, respectively) from 6 plants/treatment. Nitrogen, phosphorus, potassium, sodium, and calcium were measured according to Aslanidou et al. [25]. Briefly, the process involved an acid extraction with 6% *v*/*v* HCl of the homogenized dry tissue extracts and the subsequent use of a flame photometer for K, Na, and Ca (Jenway PFP7, Cole-Palmer, Cambridgeshire, UK). The next step for P determination was the blue-color development (ammonium vanadomolybdate/ascorbic acid method) and the absorbance measurement in a dual-beam photometer (UV1900, Shimadzu, Kyoto, Japan). As per N content, it was assessed according to the Kjeldahl method (behr Labor-Technik, Düsseldorf, Germany).

#### 4.3.3. Total Chlorophyll Content (SPAD Index)

The SPAD index (SPAD-502Plus, Konica Minolta Optics Inc., Osaka, Japan) was used to monitor the dynamics of the total chlorophyll levels of tomato leaves. A total of 4 measurements were performed during the experimental period (D12, D35, D62, and D85) on one fully developed leaf of 15 plants per treatment. All measurements were taken between 9:00 and 11:00 to ensure similar light conditions, which affect the SPAD values.

#### 4.3.4. Chlorophyll a Fluorescence In Vivo

Chlorophyll a fluorescence was measured using a FluorPen FP 110 fluorometer (PSI, Photon Systems Instruments, s.r.o., Drásov, Czech Republic), which recorded the OJIP fluorescence transients on D12, D35, and D85, between 10:00 and 11:30 a.m. For each treatment, one leaf from 15 individual plants was selected and dark-adapted for 30 min. Following dark adaptation, the leaf was exposed to a 2 s light pulse at an intensity of 3000 µmol photons m^−2^ s^−1^ with a wavelength of 650 nm. FluorPen 1.1 software was used for the OJIP parameter extraction, according to Strasser et al. [28,49] (Table 4).

#### 4.3.5. Gas Exchange

Gas exchange measurements were conducted using a portable photosynthesis system (LI-6400/XT, LI-COR, Lincoln, NE, USA). Data were collected from a single leaf on 10 plants per treatment between 9:30 and 11:30 a.m., under clear, sunny conditions on D12, D35, D62, and D85. The CO_2_ concentration in the leaf chamber was maintained at 400 ppm with the use of the 6400-01 CO_2_ Injector. The leaf chamber temperature was maintained at 25 °C, and the photosynthetic photon flux density was set to 800 μmol m^−2^ s^−1^ using the LED lamp 6400-02B (LI-COR). The following physiological parameters were recorded—net photosynthetic rate (A_n_, µmol m^−2^ s^−1^), stomatal conductance (g_s_, mol m^−2^ s^−1^), and transpiration rate (T_r_, mmol m^−2^ s^−1^)—while the intrinsic water use efficiency (iWUE, µmol mmol^−1^) was calculated as the ratio of A_n_ to T_r_ of each leaf.

### 4.4. Resource Use Efficiency Metrics

The water use efficiency (WUE) of all treatments was determined by dividing the total fruit produced (in kg) by the total volume of water used (in m^3^) during the growing cycle. Similarly, the FUE was estimated as the total fruit yield (kg) divided by the total fertilizer consumption (kg) throughout the experimental period. FUE was only considered for the HP and DCAP treatments, as the CAP treatment involved no fertilizers.

### 4.5. Statistical Analysis

One-way ANOVA was used for the statistical analysis of the results (*p* ≤ 0.05). Tukey post hoc tests were employed to assess the differences among treatments. In parameters where ANOVA pre-requisites were not satisfied, the nonparametric Kruskal–Wallis test was used. All the analyses were performed with the free statistical software JASP 0.18.3 (JASP Team 2021 Computer Software).

## 5. Conclusions

The present work investigated bioponics, a tri-trophic system incorporating crops, fish, and insects that feed each other. If the DCAP variant of aquaponics is to be adopted, bioponics can be a high-performance system. DCAP overcame all the limitations that characterized CAP performance while showing comparable growth, yield, and functional profiles to HP. However, the 31% increase in FUE demonstrated the sustainable characteristics that DCAP could achieve. We conclude that closing the nutritional loop in aquaponics by including insect meal emerges as a feasible alternative to conventional hydroponics, driving advancement in sustainable farming.

## Figures and Tables

**Table 1:** The profile of the physicochemical parameters of the three irrigation solutions, Avg ± SE. All concentrations are expressed in mmol L^−1^, with EC in dS m^−2^. The different letters in each column indicate statistically significant differences among treatments.

	pH	EC	NO_3_^−^	PO_4_^3−^	K^+^	Ca^2+^	Na^+^
HP	5.70 ± 0.03 ^b^	2.54 ± 0.07 ^a^	12.99 ± 0.32 ^a^	1.07 ± 0.04 ^a^	6.09 ± 0.21 ^a^	4.04 ± 1.00 ^a^	1.61 ± 0.06 ^a^
CAP	6.17 ± 0.20 ^a^	1.15 ± 0.03 ^b^	6.22 ± 0.48 ^b^	0.64 ± 0.10 ^b^	0.05 ± 0.03 ^b^	0.22 ± 0.01 ^b^	0.13 ± 0.00 ^b^
DCAP	5.78 ± 0.06 ^ab^	2.41 ± 0.03 ^a^	12.54 ± 0.52 ^a^	1.12 ± 0.05 ^a^	6.67 ± 0.05 ^a^	4.07 ± 0.08 ^a^	1.70 ± 0.05 ^a^

**Table 2:** WUE and FUE of tomato cultivation in the three treatments. No fertilizers were utilized in the CAP treatment; thus, no FUE value is presented.

	WUE (kg Tomato m^−3^ Water Used)	FUE (kg Tomato kg^−1^ Fertilizers Used)
HP	14.82	11.17
CAP	11.27	
DCAP	15.91	16.30

**Table 3:** Irrigation solution formula for HP tomato, according to Savvas et al. [48].

Macronutrients	Concentration (mmol/L)		Micronutrients	Concentration (μmol/L)	
	Veg. Stage	Rep. Stage		Veg. Stage	Rep. Stage
NO_3_^-^	14	14.5	Fe	15	20
NH_4_^+^	0.7	1.3	B	35	40
P	1.2	1.4	Cu	0.8	0.8
K	7	6.8	Mn	5	6
Ca	5.1	5	Zn	10	12
Mg	2.4	3	Mo	0.5	0.5
S	3.6	4.5			

**Table 4:** The parameters of the chlorophyll a fluorescence extracted from the OJIP fluorescence transients according to Strasser et al. [49].

Fluorescence Parameters	
F_M_	Maximal fluorescence from a dark-adapted leaf
F_V_	Maximal variable fluorescence from a dark-adapted leaf. FV = FM − F0
F_V_/F_M_	Maximum quantum efficiency of PSII photochemistry
Vi	Relative variable fluorescence at phase I of the fluorescence induction curve
1-Vi	Measure of relative amplitude of the IP phase in OJIP transient, related to the size of the pools of final PSI electron acceptors
1/Vi	Relative measure of the pool size of final electron acceptors of PSI
ABS/RC	Absorption flux (for PSII antenna chls) per reaction center (RC)
TR_0_/RC	Trapped energy flux per RC (at t = 0)
DI_0_/RC	Dissipated energy flux per RC (at t = 0)
PΙ_TOTAL_	Performance index total for energy conservation from photons absorbed by PSII to the reduction of PSI end acceptors
PΙ_ABS_	Performance index for energy conservation from photons absorbed by PSII antenna
Sm	Normalized area above the OJIP curve

## Data Availability

The original contributions presented in this study are included in the article. Further inquiries can be directed to the corresponding author.
